# The Nursing Supplement

**Published:** 1888-05-19

**Authors:** 


### The Nursing Mirror.

All Contributions for this Supplement should be addressed "The Nursing Mirror," care of The Editor, 38, Old Jewry, E.C.

### En Passant.

A CHURCH PARADE.—The friendly societies of Reading have been holding a church parade on behalf of the Royal Berks Hospital. It was a novel sight, and the population turned out to witness it, and willingly contributed to the collecting-boxes and bags which were held out. After marching round the town the procession divided, half attending a church service, and the other half attending a meeting in the Town Hall. The total collections amounted to £87, all contributed in small coins, pennies being the most numerous.

THE LAST STAGE OF ILLNESS.—Many of our readers may be glad to hear of a nursing home for permanent invalids, and those in the last stage of illness. Such a home exists at Brownlow Road, Reading, under the charge of Miss Vincent. Patients, if they can afford it, pay two guineas a month, and private patients are sometimes taken at a higher fee. Most of the inmates are ladies who have known better days, and who are in reduced circumstances owing to their inflictions. There is a special room for cancer cases.

THE EVILS OF OPIATES.—The death of an accoucheuse from an overdose of chloroform last week, is another warning to those who can freely obtain opiates not to be too ready to indulge in their use. The victim in this case had become so habituated to the use of the drug that she could support the most enormous doses—a pint a-day for example. The Gresham lecturer on physics during his last course of lectures cautioned nurses against helping themselves to narcotics when not ordered by the doctor. Broken nights are often the lot of a nurse, but before seeking sleep by artificial means she must consider how dangerous may be the habit she is forming, and how difficult to break.

"NURSING NOTES."—This quiet little paper pursues the even tenor of its way, far apart from the wrangling and jarring which mars its more ambitious contemporaries. It is the journal which was first established in the interests of nurses, and is devoted exclusively to nursing matters. It is solely a professional paper seeking to help nurses in their efforts to self-improvement, and does not advertise, nor in any way appeal to a public audience. This month it contains excellent and practical papers on "Heart Disease and its Nursing," "Care and Nursing of the Insane," "Nursing in Workhouse Infirmaries," etc. It has also commenced a correspondence class, and a class for the preparation of pupils for the Obstetrical Society's examination. Copies may be had at 15, Buckingham Street, Strand, W.C.

BEEF-TEA.—A medical contemporary contains a letter from a surgeon on the best and most economical method of making beef-tea. The old-fashioned plan of putting a pound of beef to a pint of water is regarded by this gentleman as wasteful and extravagant. The new method is to pass your pound of beef two or three times through a mincing machine, then place it in a closed vessel with four pints of water; let it simmer in a pan of water for five or six hours. Pass it through a colander, and any fibre which remains can be pounded in a mortar and mixed with a little strained liquor; this should now be strained through the colander, and if it has been properly pounded, will pass without leaving any residue. Then you will have four pints of beef-tea instead of one; but whether you will have four times as much nourishment is another thing.

EAST LONDON NURSING SOCIETY.—This society has a splendid plan of raising funds by means of drawing-room meetings. At one such meeting held last week the sum of £62 was collected. Canon McColl, who spoke in favour of this charity, told the following beautiful story:—A foreign Princess wished to build a hospital for the sick and suffering, but had no money; she therefore gave her jewels to be sold, aud with their price a hospital was built. When all was in order, and the poor had been received into the hospital, the Princess went round the wards. From each bed came words of thanks and praise for the king's daughter; save from one bed where a dumb woman lay: she could not speak, but when she saw the Princess great tears of gratitude gathered in her eyes and ran down her cheeks; and the Princess turned to her attendants and said: "See how my jewels are given back to me again."

A NURSING HEROINE.—A memorial in marble has been erected at Southsea to the memory of the late Mrs. Fox, who recently received a military funeral by order of the Duke of Cambridge. The inscription on the memorial is as follows: "Sacred to the memory of Mrs. George Fox, wife of Quartermaster George Fox, 2nd Connaught Rangers (94th Regiment), who died at Cambridge Barracks, Portsmouth, on January 22nd, 1888, from the effects of wounds received in the action of Bronker's Spruit, Transvaal. For her heroic and unselfish conduct on that occasion in nursing the wounded—desperately wounded though she was herself—she was decorated by Her Majesty with the Order of the Royal Cross. This monument is erected to her memory as a token of affection and esteem by the officers (past and present), non-commissioned officers, and men of the 2nd Connaught Rangers. 'Well done, thou good and faithful servant' (Matt. xxv. 21)." This inscription is surmounted by the regimental crest, a crown, an elephant, the word "Seringapatam," and the "2nd Battalion the Connaught Rangers."

EVERY WOMAN A NURSE.—The Mayor of Norwich, speaking at a meeting of the St. John's Ambulance Association lately, said he thought that in every town and village in England there should be a sort of standing Vigilance Committee of Public Safety—using the term in the very best sense—consisting principally of ladies, because the work was peculiarly their own, who would be always ready to relieve those who might be wounded or injured. It was thought worth while to have a lifeboat stationed on the coast, even though for months or years its services were not needed; so it was worth the while of ladies to acquire knowledge of nursing even though during a long life they might never require it. But he imagined there were few who could go through life without sooner or later finding such skill of the greatest possible value. It might make all the difference between life and death in a serious accident if someone were on the spot competent to deal with it. All ladies who had leisure and not an unconquerable aversion to such work ought, as a duty, to put themselves under training, in order that if opportunity arose they should be able to render help to their suffering fellow-creatures. He should be glad if in every town and village in England all who had leisure and no unconquerable aversion to the work would undertake to acquire this knowledge, for it would be beneficial and of very great advantage to the country. Women's education is indeed undergoing a great and rapid change, when they are all to be taught the rudiments of such a useful art as nursing. Musicians are trying—with success—to kill the theory that every girl, whether musical or not, should be taught the piano; and perhaps some trained nurses may think that the mediocre musician is less likely to do harm than the amateur nurse with no talent for tending the sick.

### The National Pension Fund for Nurses.

All Communications should be addressed The Editor, The Lodge, Porchester Square, W.

### THE PENSION FUND AND ITS CRITICS.

THE promoters of the National Pension Fund ought to feel flattered at the sensation their work is creating. It has been discussed in many journals, and the *Nursing Record* is so generous as to devote six columns of its scanty space to talking about it. Certainly the notice given is not flattering. It consists chiefly of unmitigated—and we may add unscrupulous—disapproval, which does not so far observe the decencies of ordinary warfare as to avoid personalities. But surely there must be something of importance in a scheme when its opponents have to spend so much force in fighting against it! The founder of the National Pension Fund ought to present his most grateful thanks to the editress of the *Nursing Record* for giving the fund all the publicity she can. We suppose it is an editress, for the nurses of the new type who have lately gone on the war-path—whose organ we take the *Nursing Record* to be—seem to have taken for their motto, "No connexion with any member of the hospital body who is a mere man." There is a decidedly feminine flavour about the pages of the *Nursing Record*. The influence of the so-called gentler sex seems to have penetrated also into the ultra-conservative *Lancet* office; at least, the articles in that journal, from which the *Nursing Record* quotes so largely, bear strong internal evidence of being inspired by the sort of female brain which Mr. Gilbert assigns to his Princess Ida:

> "The narrow-minded pedant still believes
> That two and two make four. Why, we can prove—
> We women, household drudges as we are—
> That two and two make five, or three, or seven,
> Or seven and twenty, if the case demands."

In the present instance Mr. George King, the actuary who drew up the Pension Fund tables, is the narrow-minded pedant. The *Lancet* protests that his two and two make three—that is, that his tables are not calculated at the lowest possible rates compatible with safety. Mr. King explained the facts in a letter which the *Lancet* refused to publish—the *audi alteram partem* which is placed at the top of its correspondence column being like the motto on a parvenu's crest, a decorative device with no meaning whatsoever. But some friend has convinced the editor that his position is untenable; so next week the declaration is that two and two make five or seven, and why didn't Mr. King say so? Mr. King being a prudent man, doesn't like to promise more than he knows he can give, or to base statistics on even the best probabilities; but the *Lancet* is very angry all the same. It protests that it is very much wickeder not to tell all you can give—or more!—than to promise more than you can perform. To the average mind this appears both absurd and dishonourable, but the *Nursing Record* quotes the articles which are founded on this argument with immense satisfaction. We rather think, however, that the first article was transferred from the one journal to the other without being read, or a very moderate regard for consistency would have dictated the removal of either a sentence which the *Lancet* quotes approvingly from the *Times* or a paragraph in *Nursing Echoes*. The first runs thus: "The charitable element in the society is calculated to deal a blow to the cause of women's independence;" the second thus: "Many nurses do urgently need some provision for sickness and old age." The S.G.—by-the-bye, do these initials stand for Sairey Gamp?—who writes *Nursing Echoes*, should be a little more careful. It doesn't, it really doesn't, answer for a journal to contradict itself so obviously.

BETSY PRIG.

### WEARY OF THE SUBJECT.

As a constant reader, will you allow me space to tell an amusing anecdote, with the view of pointing a moral, if not adorning a tale? It is recorded that on one occasion the late Master of the Rolls, then Mr. Jessel, was pleading a cause before a certain learned judge, and in the course of his address he repeated, as he very frequently did, the arguments with which he was supporting his contention. This greatly irritated the learned judge, with whom Jessel was by no means a favourite, and he suddenly interrupted him with the remark: "Mr. Jessel, when a counsel in stating his case before me makes use of any argument to enforce the proposition that he is setting forth, I give to it such attention as the cogency of the argument and the eminence of the learned counsel employing it would seem to demand. Should it, however, be *repeated*, it entirely loses its effect upon me, and it is my invariable custom to dismiss it at once from my mind." "Then, my lord," replied Jessel, "the greater the necessity that I should repeat it to your lordship a *third* time;" and this he proceeded to do. We cannot but admire the ready wit of the eminent counsel; but our sympathy must rest, I think, unquestionably with the learned judge. Now, sir, will you allow me most respectfully to suggest by way of moral that the arguments in the case of the "National Pension Fund *versus* the *Lancet*" have been stated and insisted upon almost *ad nauseam* in THE HOSPITAL. No one with one grain of candour in his composition can for a moment doubt on whose side justice lies; and I cannot help thinking that Mr. Burdett only weakens his case by its constant reiteration; and the question has a tendency to sink to the level of a petty personal squabble. The character of Mr. Burdett has been so strong, and his work of so beneficent a nature that we are almost disappointed that he should allow himself to be seriously disturbed because a purely medical journal fails to deal accurately with a question of figures. Surely, sir, you are not wanting subjects of general interest that you need fall back so continually on this. With many apologies, I am, &c., W. ASHE PAYNE.

### Workhouse Nursing.

THE Workhouse Infirmary Nursing Association, which advertises constantly in our pages for nurses, seems to interest our readers so greatly that we propose to give a few particulars concerning it. The Association was begun in 1879, to improve the nursing in workhouse infirmaries, and to promote the employment of trained nurses and matrons in these institutions. Ever since its commencement the Association has been steadily growing, till it has now at work in different infirmaries, nearly a hundred nurses. During 1887 thirty-three nurses were appointed to twenty-two infirmaries, many of the nurses having been trained at the expense of the Association. Most of these nurses are engaged in London, it being very difficult to persuade country Boards of Guardians to properly treat and pay a trained nurse; and also difficult to persuade nurses to undertake onerous duties in an isolated situation.

The following are the rules for probationers who join the association:—

1.—The usual term of training will be a year.

2.—At the end of a year probationers will be provided with situations by the association, and for the next three years (at least) they will be required to take such posts as the committee will offer them, whether in London or the country, and on day or night duty.

3.—If any nurse shall leave the service of the Association during the three years following her training, she shall be liable to repay the sum expended by the Association on her training.

4.—When the probationer has completed her training, and has remained one year in a situation with satisfaction to the authorities of the infirmary, she will receive a medal, which he is required to return to the hon. secretary, should she for any cause leave the service of the Association.

5.—No nurse probationer is permitted to give notice of leaving any situation without permission from the hon. secretary.

6.—All certificates from institutions are to be sent to the hon. secretary, and not to be retained by the nurse probationers.

The rules for nurses are as follows:

1.—Nurses who remain in a situation to which they have been recommended by the Association, and in which they give satisfaction to the authorities of the infirmary, will receive a gratuity for a second year in a situation £1 10*s*., for the third and every following year £2, for a year's service in a new situation £1.

2.—The medal of the Association will be given at the end of two years' good service. The gratuity and medal will be given at the annual meeting of the nurses in the month of May of each year.

3.—Nurses are required to give notice to the hon. secretary of any intention of leaving a situation, and they will be required to return the medal, if for any cause they give up their connexion with the Association.

4.—It is hoped and expected that every nurse will make some provision for the future by placing a sum quarterly in the Post Office Savings Bank.

It will be seen from these rules that one great trouble is to persuade nurses to remain steadily at their posts till early difficulties are overcome. It really needs a self-sacrificing spirit to take up the work formerly done—or left undone—by an incompetent pauper, and to reduce all to method and cleanliness. There is often a lack of the simplest appliances of nursing, such as thermometers, bandages, &c., and one nurse who asked to be allowed a screen was regarded in astonishment as a creature of the most absurd super-refinement. Paupers are not supposed to have any feelings which should be respected, nor trained nurses to have any modesty. Then the nurse will often find her room small and dark, and find also that she is expected to cook her dinner in the one room which acts as both bed-room and sitting-room. Visitors to workhouse infirmaries are also rare; the guardians seldom encourage them, and the nurse finds that often a week goes by without an opportunity offering to exchange a few sentences with a single person of her own station. All these troubles pass with time if the nurse only exercise sufficient patience and tact, but it is very evident that only a noble and devoted woman is equal to the task of first occupying the position of trained nurse in an out-of-the-way workhouse where a pauper inmate preceded her. I remember going to see an infirmary in the Midlands with the labour-mistress of the workhouse. She led me to the detached building set aside for the sick, and, entering a lobby, unlocked a door on the right hand. We entered a long ward containing twenty beds, half of which were occupied by women. Two women, one nursing a baby only a fortnight old, were sitting by the fire. The place was neat and clean, but had a cheerless aspect. We went out into the lobby again, and the mistress unlocked the door on the left-hand side, and we entered a similar ward only occupied by men. "But where are the nurses?" I asked, looking round in wonderment, for except an old man wandering about with a mug, I could see only the patients in bed. "There is only one nurse, and she has gone out for a walk," coldly replied the mistress. It was a new experience to me to learn that one nurse could have charge of forty beds both day and night, and when she wanted exercise and fresh air could lock up her patients and go for a walk. These are the evils against which the Association is fighting, and if its want for country infirmaries is good trained nurses, its want for town infirmaries is lady matrons and lady guardians. If there were a few more educated and enlightened women willing to act as guardians, we should not so frequently have paragraphs like the following in the papers:—

"Pauper Nursing.—Last night, at a meeting of the Holborn Board of Guardians, Mr. Pedder asked for a return of the nursing staff at the Highgate Infirmary. There were fifteen wards, with an average of seventy-five patients in each, and only one night nurse for two wards. Mr. Langley: A man complained to me that there were helpless patients in one of the wards who had not been fed or washed for four days. ('Oh, oh!') Mr. Miller: I emphatically deny that. No pauper ever goes a day without washing. Mr. Pedder further said that two patients died in one ward within twenty minutes of each other, so they could have had but little attention from the one nurse. The Clerk said that for the fifteen wards there were eight 'charge' nurses, eighteen assistant nurses, and two temporary nurses. It was resolved to consider as to appointing more assistance. Mr. Jacobs remarked that whilst the Board were considering this matter they might pay some attention to Gray's Inn Road, where there were 122 lunatics, imbeciles, and sick in seven wards, and cared for by only three nurses. Indeed, only one nurse was in charge of the whole number during the night. This also was referred to the committee."—*Daily News*, March 11th.

Unless there is a lady on the Board it is almost impossible to make the guardians understand the necessity and economy of having trained nurses to attend to the sick, and of supplying these nurses with all proper appliances for the thorough fulfilling of their work.

Probationers are trained by the Association at Brownlow Hill, Manchester, and other infirmaries. The training costs from £10 to £15, and if during the next three years the nurse leaves the Association, she must repay this money. During her year of training a nurse receives a salary of £5 to cover her incidental expenses. Hospital training is not well suited for union work, for after the excitement of operations, surgical work, &c., nurses find their duties in county infirmaries, amongst chronic and bedridden cases, very dull. London is easier to supply, but the provincial infirmaries are most in need of improvement. The gratuities have been given to induce nurses to remain steadily in one situation, and have been very helpful in that respect. When the Association first started they had ceaseless resignations, for nurses would not put up with the trials and discomforts which often assailed them. Since the giving of gratuities was commenced, the proportion of resignations has been much smaller, and the nurses are doing better and steadier work; feeling that they are going to remain in one place, they devote their energies to improving their surroundings, often with the greatest success. It is a pity that the gratuity which used to be given at the conclusion of the first year of service has had to be abandoned, owing to the Association suffering from want of funds; from the same reason the Association cannot train so many probationers as it would like.

### Lectures.

A course of six lectures on "Dante," by the Rev. E. Moore, Barlow lecturer, will be commenced at University College at three p.m., on May 16th, free to the public.

### Everybody's Opinion.

MORE ABOUT PROBATIONERS.

To the protest of "Nurse V." might the following be added? A well-known London institute undertakes to supply a Yorkshire infirmary with nurses. The ordinary staff are a matron, head nurse, and three probationers. There are 27 beds, and the probationers have often to take entire charge and night duty alone. When there is an infectious case one of them is isolated, and besides the nursing she has to scrub the floors and clean the grates, or go with them dirty, as long as the case lasts, which has been over eight weeks. They are bound to serve the institute three years at £14, £17, and £20 per annum, including the training year. Partial uniform is provided. This is only one; there may be others the same. Is it being paid for learning? S. M. ROSE.

### Appointments.

MISS GERTRUDE VACHER has been appointed matron of the Eccles and Patricroft Hospital in place of Miss Morris, who has resigned. Miss Vacher has lately concluded her period of training at the London Hospital, and for the last six months has had charge of a women's surgical and accident ward. Miss Vacher took the first prize at last year's examination of probationers at the London Hospital, and her certificate is a remarkably good one.

MISS MACDONNELL has been appointed lady superintendent of Richmond Hospital, Dublin; and Miss Hughes has been appointed superintendent of Whitworth Hospital, Dublin. Both ladies were trained at Sir Patrick Dun's Hospital.

### Vacancies.

The following vacancies are announced:—

**Matron.**

Kingston Cottage Hospital; no salary.

**Nurses.**

National Hospital, Queen Square, Two; £25; £20.

Oakfield, Forest Hill.

Children's Hospital, Cardington, near Bedford.

Royal Surrey County Hospital: £25.

Nurses' Home, Truro.

Newark Nurses' Institute; £20.

Eye and Ear Hospital, Bradford.

Burton-on-Trent Nursing Institution; £25 to £28.

**Probationers.**

Burton-on-Trent.
Infirmary for Children, Sydenham.
Wimborne Cottage Hospital.

**District Nurses.**

Petersfield.
Chester.

**Ward-maid.**

Hammerwich Cottage Hospital.

### Examination Questions.

The following questions were given to probationers in a provincial hospital:—

(1) What are the meanings of the following words? Cataplasm, sinapism, hirudines, clyster, haustus, stupe and balneum tepideum, calidum, frigidium?

(2) How should you read the following terms? Oli ricini, oli morrhuæ, haust sennæ co., vin ipecacuanhæ, pulveris sinapis, cup i sulphatis, zinc sulphatis.

(3) What is a collcyrium, colentorium, decoctum, gargarisma, linimentum, lotio, unguentum, emplastrum.

(4) What is an emetic, aperient, anodyne, diuretic, diaphoretic, drastic, narcotic, soporific, sudorific, astringent, styptic, tonic, anthelmintic, emmenagogue, emollient, expectorant, refrigerant, and a sedative.

### Notes and Queries.

TO CORRESPONDENTS.—1. Questions may be written on post-cards. 2. Advertisements in disguise are inadmissible. 3. In answering a query please quote the number. 4. If a private answer is desired, a stamped addressed envelope must be forwarded.

12.—Could I be informed through the medium of your paper if there is any hospital or training school where trained but non-certificated nurses in private work might enter their names for examination? N.M.R.—Can any reader give the desired information?

13.—Would you kindly tell me the kind of place the Hospital at Ventnor is, if it is a large one? Nurse Faith.—The Ventnor Hospital for Consumption, contains 140 beds.

14.—Will the Editor inform "Nurse," through the medium of THE HOSPITAL "Notes and Queries," where massage can be learned?—Application may be made to Secretary, 14, Welbeck Street, W.

15.—Can you suggest anything, besides strict personal cleanliness and the constant use of "Keating's Powder," which will ward off the attacks of that troublesome little creature, the bug? And when it has left its disfiguring mark upon the long-suffering individual, is there nothing to alleviate the irritation and swelling?—Can any sympathiser make a suggestion.—ED.

ADDRESS WANTED.—A letter has been sent to THE HOSPITAL Office for Miss Rose Macbride. It will be forwarded on receipt of her address.

**Answers.**

(12) *Erin* is recommended to apply to the New Hospital for Women, 222, Marylebone Road; or to Mrs. Meredith's dispensary, Clapham Road.

*Miss Lewis*, Crewe.—Your letter has been forwarded to Mrs. Bedford Fenwick, the founder of the British Nurses' Association, with the request that she will send you the desired information.

### Exchange and Mart.

UNDER this heading we propose to insert purely Exchange advertisements, at a charge of sixpence for eighteen words, or three insertions for one shilling. It must be distinctly understood that ordinary advertisements will not be inserted here.

RENSHAWS MANUALS. — "Anatomy," Malgaine (12s 6d.), "Elementary Botany, Dr. Silver (12s. 6d.), "Human Osteology" Ward (7s.) Would exchange either for Nurse's Surgical dressing case. What offer? Also Owen's "Materia Medica," "A Guide to the New Pharmacopœia" "First Book of Botany, Balfour. The last three for 5s.; or one guinea the lot. All in good condition. Address C., THE HOSPITAL Office, 38, Old Jewry, E.C.

### MR. BURDETT'S BOOKS ON HOSPITALS.

"Mr. Henry C. Burdett, whose interest in Hospitals is well known, is widely recognised as the chief authority on the subject."—*The Lancet*, July 25th, 1885.

**HOSPITALS AND THE STATE: HOSPITAL MANAGEMENT, AND HOSPITAL NURSING.** With Statistics of the Chief Medical Institutions in Great Britain and Ireland. [Cloth, 2s. 6d.

*Boston Medical and Surgical Journal.*—"Mr. Burdett is one of the highest authorities of Great Britain on the subject of Hospital Administration."

*British Medical Journal.*—"The only accurate statistics of hospital income and expenditure, prepared upon an identical basis, which have ever been published."

**COTTAGE HOSPITALS: GENERAL, FEVER, AND CONVALESCENT.** Their Progress, Management, and Work. With an Alphabetical List of every Cottage Hospital at present opened, and Chapters on Mortuaries, Convalescent Cottages, and the Relative Mortality in Large and Small Hospitals. The book contains the ground-plan and elevation of the best constructed British Hospitals and Medical Institutions having fifty beds and under, and also a Portrait of Albert Napper, Esq., the Founder of Cottage Hospitals. Second Edition. 600 pp. [Cloth, 14s.

**PAY HOSPITALS AND PAYING WARDS THROUGHOUT THE WORLD:** Facts in support of a Re-arrangement of the English System of Medical Relief. 200 pp. [Demy 8vo., 6s.

**SELECTED EXTRACTS FROM REVIEWS.**

*British Medical Journal.*—"Mr. Burdett is well known to the medical profession as an advocate of provident dispensaries and hospitals, and as the originator of the Home Hospitals Association. All who are interested in these subjects will be gratified by this volume. In it the author deals with the whole question of pay hospitals and dispensaries in a large and comprehensive spirit. We commend the book to all who are interested in the improvement of medical relief—and which of us is not? It is clearly and pleasantly written, it is full of facts, and it contains many valuable suggestions. This work cannot fail to stimulate the reforming movements which have been gathering strength in this country during the last few years."

*New York Medical Journal.*—"Mr. Burdett has devoted many years to the study of hospital administration and management, and his book and plans are well worth perusal."

*The American Practitioner.*—"Mr. Burdett displays and discusses the whole scheme of hospital accommodation with a comprehensive understanding of its nature and extent, and he does it in fulness without prolixity, and in a clear catholic spirit with perspicacity. The book is a stepping-stone, a valuable contribution in the way of introduction to a review and candid reconsideration of the whole subject of legal and organised charity—a theme which much demands consideration and re-adjustment in the whole civilised world. A good and timely book, and suggestive."

**London: J. and A. CHURCHILL, 11, New Burlington Street, W.**

